# Artificial Intelligence–derived Measurements of Myosteatosis from Coronary Artery Calcium CT Scans to Predict COPD: The Multi-Ethnic Study of Atherosclerosis

**DOI:** 10.1148/ryct.250205

**Published:** 2026-01-29

**Authors:** Amir Azimi, Kyle Atlas, Anthony P. Reeves, Chenyu Zhang, Jakob Wasserthal, Seyed Reza Mirjalili, Thomas Atlas, Claudia I. Henschke, David F. Yankelevitz, Javier J. Zulueta, Juan P. de-Torres, Luis M. Seijo, Jeffrey I. Mechanick, Andrea Branch, Ning Ma, Rowena Yip, Wenjun Fan, Sion K. Roy, Khurram Nasir, Sabee Molloi, Zahi A. Fayad, Michael V. McConnell, Ioannis A. Kakadiaris, George S. Abela, Rozemarijn Vliegenthart, David J. Maron, Jagat Narula, Kim A. Williams, Prediman K. Shah, Matthew J. Budoff, Daniel Levy, Emelia J. Benjamin, Roxana Mehran, Robert A. Kloner, Nathan D. Wong, Morteza Naghavi

**Affiliations:** ^1^HeartLung.AI, 2450 Holcombe Blvd, Houston, TX 77021; ^2^Department of Electrical and Computer Engineering, Cornell University, Ithaca, NY; ^3^Clinic of Radiology and Nuclear Medicine, University of Basel, Basel, Switzerland; ^4^Tustin Teleradiology, Tustin, Calif; ^5^Department of Radiology, Icahn School of Medicine at Mount Sinai, New York, NY; ^6^Pulmonary Department, Clínica Universidad de Navarra, Pamplona, Spain; ^7^Clínica Universitaria de Navarra, Pamplona, Spain; ^8^Kravis Center for Clinical Cardiovascular Health, Mount Sinai Fuster Heart Hospital, New York, NY; ^9^Department of Radiology, University of California Irvine, Irvine, Calif; ^10^The Lundquist Institute, Torrance, Calif; ^11^Health Equity and Disparities Research, Division of Cardiovascular Prevention and Wellness, and Center for Cardiovascular Computational Health & Precision Medicine (C3-PH), Houston Methodist Hospital, Houston, Tex; ^12^Stanford Prevention Research Center, Stanford University School of Medicine, Stanford, Calif; ^13^University of Houston, Houston, Tex; ^14^University of Louisville, Louisville, Ky; ^15^Cedars-Sinai Medical Center, Los Angeles, Calif; ^16^Division of Cardiology, Michigan State University, East Lansing, Mich; ^17^Department of Radiology, University Medical Center Groningen, Groningen, the Netherlands; ^18^School of Public Health, Boston University, Boston, Mass; ^19^Department of Cardiovascular Medicine, Huntington Medical Research Institutes, Pasadena, Calif; ^20^Heart Disease Prevention Program, Mary and Steve Wen Cardiovascular Division, University of California Irvine, Irvine, Calif

**Keywords:** Applications-CT, Pulmonary, Thorax, Adipose Tissue (Obesity Studies), Chronic Obstructive Pulmonary Disease, Metabolic Disorders, Myosteatosis, Coronary Artery Calcium Scan, Emphysema, AI-CVD

## Abstract

**Purpose:**

To evaluate the predictive value of myosteatosis as an opportunistic finding in coronary artery calcium (CAC) CT scans for clinically diagnosed chronic obstructive pulmonary disease (COPD) and compare it with an artificial intelligence (AI)–measured biomarker of emphysema derived from the same scans.

**Materials and Methods:**

In this prospective study, baseline CAC CT scans and 20-year follow-up data were analyzed. Myosteatosis was defined as the lowest quartile of thoracic skeletal muscle mean attenuation (males < 33.5 HU, females < 27.0 HU). The emphysema-like lung biomarker was quantified as the percentage of lung voxels below −950 HU in CAC CT scans. COPD was identified using the *International Classification of Diseases, Ninth Revision, Clinical Modification*, and *International Classification of Diseases, 10th Revision, Clinical Modification* diagnostic codes from hospital discharge records. Hazard ratios (HRs) for COPD were calculated using proportional hazard regression models, comparing the bottom versus top quartiles of myosteatosis and emphysema-like lung measurements.

**Results:**

Among 5535 participants in the Multi-Ethnic Study of Atherosclerosis (mean age ± SD, 62.2 years ± 10.3, 47.6% males), 396 (7.1%) were diagnosed with COPD over the 20-year follow-up period. Myosteatosis showed a stronger association with COPD than emphysema (unadjusted HRs, 5.98 [95% CI: 4.14, 8.63] and 2.12 [95% CI: 1.61, 2.78], respectively [*P* < .001]). After adjusting for covariates (age, sex, smoking status, body mass index, race, asthma, physical activity, inflammatory markers, and insulin resistance), the HRs were reduced to 2.74 (95% CI: 1.81, 4.16) and 1.50 (95% CI: 1.12, 2.00), respectively (*P* = .02).

**Conclusion:**

AI-measured myosteatosis in CAC CT scans strongly predicted future diagnosed COPD independently of known risk factors.

**Keywords:** Applications-CT, Pulmonary, Thorax, Adipose Tissue (Obesity Studies), Chronic Obstructive Pulmonary Disease, Metabolic Disorders, Myosteatosis, Coronary Artery Calcium Scan, Emphysema, AI-CVD

ClinicalTrials.gov: NCT00005487

*Supplemental material is available for this article.*

© The Author(s) 2026. Published by the Radiological Society of North America under a CC BY 4.0 license.

## Graphical abstract

**Figure:**
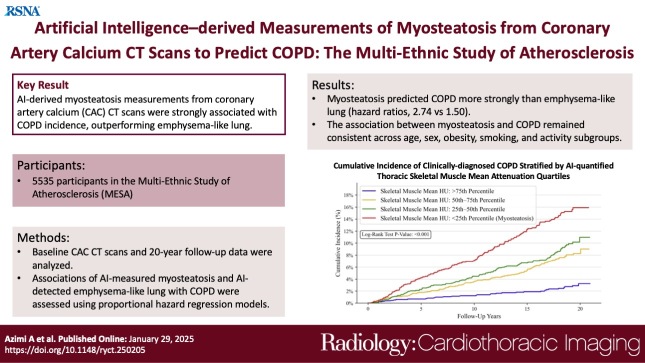

**Summary**

Artificial intelligence–derived myosteatosis measurements from coronary artery calcium CT scans were strongly associated with the incidence of clinically diagnosed chronic obstructive pulmonary disease, outperforming emphysema-like lung as a predictive biomarker.

**Key Points**

■ In a prospective study involving 5535 participants, artificial intelligence (AI)–derived measurements of myosteatosis from coronary artery calcium CT scans predicted clinical chronic obstructive pulmonary disease (COPD) incidence; the lowest-quartile muscle quality conferred a 2.74-fold higher risk than the highest quartile.
■ Myosteatosis predicted COPD more strongly than AI-detected emphysema-like lung (hazard ratio, 2.74 vs 1.50; *P* = .02).
■ The association between myosteatosis and COPD remained consistent across age, sex, obesity, smoking status, and activity subgroups.

## Introduction

Chronic obstructive pulmonary disease (COPD) is a preventable, chronic inflammatory lung condition characterized by irreversible airflow limitation and remains a leading cause of morbidity and mortality worldwide (1,2). Despite the identification of environmental and genetic risk factors, substantial gaps remain in understanding COPD pathogenesis, highlighting the critical need for novel predictive biomarkers to enable early detection and targeted prevention, particularly in traditionally low-risk populations (1).

Myosteatosis, characterized by excessive fat infiltration within skeletal muscle tissue, has emerged as a critical indicator of compromised muscle quality that extends beyond simple measures of muscle mass or strength (3). This pathologic process involves both inter- and intramyocellular lipid accumulation resulting from aging, physical inactivity, and metabolic dysregulation, particularly insulin resistance (4). The condition can be reliably quantified through muscle radiodensity measurements obtained from noncontrast CT scans, demonstrating a strong correlation with histologic biopsy findings (5). Beyond its well-documented associations with diabetes, obesity, and diminished physical function, myosteatosis appears to influence systemic health through distinct pathophysiologic mechanisms (5,6).

The systemic implications of myosteatosis extend beyond localized muscle dysfunction because intramuscular fat deposits function as metabolically active tissue that promotes proinflammatory cytokine production and contributes to sustained systemic inflammation (4). This inflammatory cascade, combined with associated mitochondrial dysfunction and insulin resistance that characterize myosteatosis, creates a pathophysiologic environment remarkably similar to established COPD risk factors (7). Prior studies have demonstrated that systemic inflammation, metabolic disorders, mitochondrial dysfunction, and insulin resistance independently predict the development of COPD. The presence of myosteatosis, therefore, may represent an early mechanistic pathway that precedes and potentially contributes to COPD pathogenesis (7–9).

Opportunistic screening represents an innovative approach for early detection of chronic diseases and risk stratification, leveraging incidental information embedded within imaging studies performed for unrelated clinical indications (10). The integration of artificial intelligence (AI) has revolutionized the analysis of these opportunistic imaging findings, transforming manual processes into systematic, reproducible assessment tools. Our research group developed the AI cardiovascular disorders (AI-CVD) platform to maximize the clinical value of routine CT scans, particularly coronary artery calcium (CAC) scans, by extracting both coronary and noncoronary findings to enhance chronic disease prediction across multiple conditions (11). Our group has previously demonstrated that AI-measured myosteatosis in thoracic skeletal muscles (TSMs) from CAC CT scans independently predicts cardiovascular outcomes, particularly atrial fibrillation and heart failure, in the Multi-Ethnic Study of Atherosclerosis (MESA) (12). The AI-CVD platform comprehensively analyzes CAC CT scans, with previous publications validating several components, including automated measurements of bone mineral density, cardiac chamber volumes, and left ventricular mass (13–17).

Previous investigations of muscle abnormalities in COPD have been limited by critical methodologic constraints that have hindered the understanding of the temporal relationship between changes in muscle composition and disease development. Most research has focused on patients with established COPD, making it impossible to determine whether muscle changes precede or result from disease onset (18–20). Additionally, prior studies have typically examined isolated muscle groups or single-section measurements rather than comprehensive thoracic muscle assessment, potentially overlooking the systemic nature of muscle quality deterioration. The predominant use of cross-sectional designs has further limited the ability to establish temporal relationships between muscle composition and COPD development (21). In this study, we investigated whether AI-quantified myosteatosis in TSMs predicts the incidence of clinically diagnosed COPD risk over 20 years in MESA participants and compared its predictive value with that of AI-detected emphysema-like lung in the same scans. This analysis aimed to expand our understanding of myosteatosis as a potential early biomarker for COPD development in a community-based cohort.

## Materials and Methods

### Study Design and Sample

This analysis used data from the MESA (ClinicalTrials.gov NCT00005487), a prospective cohort study initiated in July 2000 with follow-up through July 2020. The MESA was designed to investigate the prevalence, correlates, and progression of subclinical cardiovascular disease in individuals without known cardiovascular disease at baseline. The study recruited 6814 participants, both female and male, aged 45–84 years, from six diverse U.S. communities: Baltimore (Maryland), Chicago (Illinois), Forsyth County (North Carolina), Los Angeles County (California), northern Manhattan (New York), and St. Paul (Minnesota). A comprehensive description of the MESA study design has been previously published (22).

### Ethical Approval

The MESA is a longitudinal population-based study sponsored by the National Institutes of Health and has received proper ethical oversight. The MESA protocol was approved by the institutional review boards of the six field medical centers (Columbia University, Johns Hopkins Medicine, Northwestern University, University of California, Los Angeles, University of Minnesota, and Wake Forest) and the National Heart, Lung, and Blood Institute. Written informed consent was obtained from all participants for imaging and linkage to clinical outcomes.

### Participant Selection

**Figure 1:**
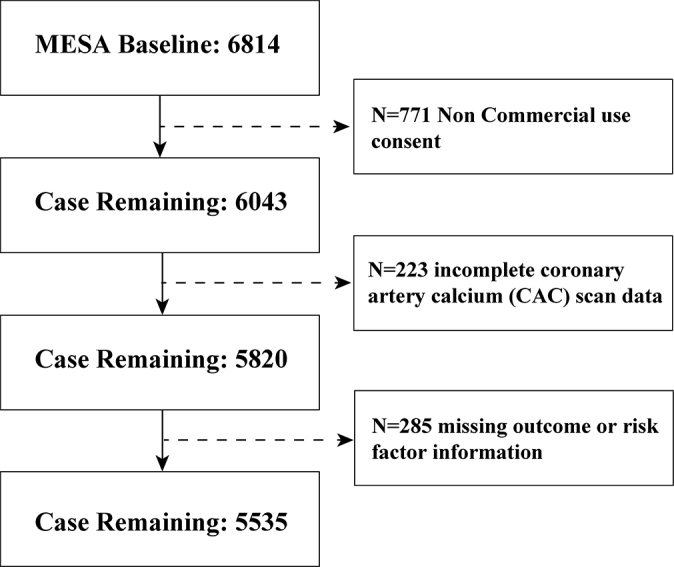
Flow diagram shows study sample inclusion and exclusion. MESA = Multi-Ethnic Study of Atherosclerosis

Several exclusion criteria were implemented to establish the analytical sample from the initial MESA cohort. Overall, 771 participants were excluded due to nonconsent for commercial use of their data, 222 due to incomplete CT scan data (missing sections), and 286 due to missing outcome or risk factor information. After applying these exclusion criteria, our final analytic sample consisted of 5535 participants (Fig 1).

Clinically diagnosed COPD was identified through hospital discharge records using *International Classification of Diseases, Ninth Revision, Clinical Modification* and *International Classification of Diseases, 10th Revision, Clinical Modification* diagnostic codes. *International Classification of Diseases, Ninth Revision, Clinical Modification* codes included chronic bronchitis (491.0, 491.1, 491.20, 491.21, 491.22, 491.8, 491.9), emphysema (492.0, 492.8), and chronic obstructive airway disease (496). *International Classification of Diseases, 10th Revision, Clinical Modification* codes encompassed chronic bronchitis (J40, J41.0, J41.1, J41.8, J42), emphysema (J43.0-J43.9), and other COPDs (J44.0, J44.1, J44.9). Only hospitalized events with these diagnostic codes were considered because outpatient events were not captured in the MESA database. The time to COPD diagnosis was calculated in days from the baseline examination to the first occurrence of a qualifying COPD diagnosis. Participants were censored at death, loss to follow-up, or end of the study period.

### AI Analysis for Myosteatosis and Emphysema Assessment with Cardiac CT

Myosteatosis evaluation was performed using AI-CVD (HeartLung.AI), a specialized deep learning platform designed for comprehensive opportunistic analysis of anatomic structures in cardiac and chest CT scans, with capability for both contrast-enhanced and noncontrast imaging (Appendix S1). In this study, myosteatosis evaluation was performed with noncontrast cardiac CT. Myosteatosis was operationally defined as measurements in the lowest quartile of TSM mean attenuation Hounsfield units, with sex-specific thresholds established at less than or equal to 27.0 mean HU for females and less than or equal to 33.5 mean HU for males. The AI model segmented all visible TSMs across the entire scan volume, rather than relying on a single axial section or fixed region of interest. Specifically, muscle volume and mean attenuation (in Hounsfield units) were derived from the cumulative segmentation of all thoracic cavity muscles, including the pectoralis major and minor, paraspinal muscles (erector spinae and multifidus), and intercostal muscles, within the CAC CT scan field.

Additionally, we measured emphysema-like lung in the same CAC CT scans to evaluate its association with clinically diagnosed COPD compared with that of myosteatosis. Regions of the lung parenchyma with attenuation values below −950 HU are a known radiologic surrogate for emphysema-like lung (23). We applied AI-CVD software to segment and analyze all five lung lobes from CAC CT scans. The mean lobe volumes ± SD were as follows: upper left, 437.0 mL ± 160.0; lower left, 773.0 mL ± 251.1; upper right, 212.6 mL ± 124.9; middle right, 377.3 mL ± 119.5; and lower right, 901.9 mL ± 270.6. Subsequently, we calculated the percentage of total lung voxels below −950 HU for further analysis.

The fundamental machine learning architecture of AI-CVD was derived from TotalSegmentator, a validated anatomic modeling system developed by independent investigators and coauthors of this manuscript (24). The source code for TotalSegmentator is available publicly from GitHub *(https://github.com/wasserth/TotalSegmentator)*.

To address potential confounding effects of paraseptal emphysema, which affects distal acini and subpleural and/or paraseptal regions but is not associated with COPD (25), we conducted a sensitivity analysis. In 100 randomly selected cases, we digitally eroded 20% from the periphery of each lung lobe in the emphysema calculation. Comparison of emphysema percentages before and after this removal showed no statistically significant differences, suggesting our findings are robust to the potential diluting effects of paraseptal emphysema.

### Statistical Analysis

All statistical analyses were performed using R Studio version 4.5.1 *(https://posit.co/download/rstudio-desktop/)* and Python version 3.10 *(https://www.python.org/)*. All tests of significance were two-tailed, with type I error set at α equals .05. For normally distributed continuous variables, data were presented as means ± SDs, and comparisons were conducted using independent *t* tests for two groups and one-way analysis of variance for multiple groups. Nonnormally distributed continuous variables were presented as medians with IQRs, with group comparisons performed using the Wilcoxon rank sum test for two groups and the Kruskal-Wallis test for multiple groups. Categorical variables were expressed as frequencies with percentages and analyzed using χ^2^ tests.

The association between myosteatosis and the incidence of clinically diagnosed COPD was evaluated using Cox proportional hazards regression, analyzed categorically in quartiles and continuously using restricted cubic splines with knots placed at evenly spaced quantiles. The proportional hazards assumption was tested between myosteatosis and the incidence of clinically diagnosed COPD using Schoenfield residuals, and no violations were detected. The analysis incorporated a hierarchical adjustment approach based on known COPD risk factors, beginning with an unadjusted model (model 1), followed by sequential adjustment for age (model 2), additional demographic factors including sex, body mass index (calculated by dividing weight in kilograms by height in meters squared), and race (model 3), pack-year smoking (model 4), asthma (model 5), physical activity (model 6), inflammatory indexes (C-reactive protein and interleukin 6) (model 7), and insulin resistance (model 8). Insulin resistance was estimated using the homeostasis model assessment of insulin resistance, calculated as fasting insulin (mIU/L) × fasting glucose (mg/dL)/405. Homeostasis model assessment of insulin resistance was natural logarithm (ln) transformed due to positive skewness. Physical activity was calculated using metabolic equivalent weekly task hours for moderate exercise. Race and ethnicity variables were one-hot encoded with the White group serving as the reference category, and the Chinese, Black, and Hispanic groups were compared against the White group. We also evaluated emphysema-like lung measurements, although in contrast to myosteatosis, we compared the highest quartile versus the lowest quartile. The relative strength of association between myosteatosis and emphysema-like measurements with clinically diagnosed COPD was compared using Wald tests across all models.

The cumulative incidence of clinically diagnosed COPD by quartiles of TSM mean attenuation was calculated using the one minus the Kaplan-Meier survival estimate. Group differences were evaluated using the multivariate log-rank test.

Subgroup analyses were conducted based on age at recruitment (<60, ≥60 years), sex (male, female), body mass index (<30, ≥30), smoking status (<20, ≥20 pack-year smoking), physical activity (<median, ≥median), homeostasis model assessment of insulin resistance (<2.5, ≥2.5), emphysema-like lung percentage (<median, ≥median), and passive smoking exposure (ever, never). Passive smoking was assessed exclusively in participants who reported never smoking or who had formerly smoked. Sensitivity analyses were performed by excluding participants who were diagnosed with COPD within the first 2 years of follow-up and those with asthma at baseline.

To assess whether the myosteatosis–COPD association varied by age or smoking exposure, we conducted a Cox proportional hazards analysis with interaction terms. TSM mean attenuation, age, and pack-years were each mean-centered, and two interaction terms (attenuation × age; attenuation × pack-years) were created. These, plus main effects and covariates, were entered into the fully adjusted model. We evaluated each interaction via Wald tests and their joint contribution via a likelihood-ratio test, using two-tailed α equals .05.

## Results

### Participant Characteristics

This study analyzed 5535 participants (mean age ± SD, 62.2 years ± 10.3) (Fig 1). The cohort included 2633 male (47.6%) participants and 2817 participants who had never smoked (50.9%). For race and ethnicity, 2186 participants were non-Hispanic White (39.5%), 1428 were Black (25.8%), 1228 were Hispanic (22.2%), and 693 were Chinese American (12.5%). Baseline characteristics stratified by the highest and lowest TSM mean attenuation quartiles are shown in Table 1.

**Table 1:** Baseline Characteristics of the Overall Cohort between the Top versus Bottom Quartile of AI-quantified TSMMean Attenuation in CAC CT Scans

Characteristic	Total Population (*n* = 5535)	Top Quartile of AI-quantified TSM Mean Attenuation (*n* = 1360)	Bottom Quartile of AI-quantified TSM Mean Attenuation (Myosteatosis) (*n* = 1396)
Age (y)	62.19 ± 10.31	55.68 ± 8.23	67.61 ± 9.80
Male sex	2633 (47.6)	646 (47.5)	662 (47.4)
Female sex	2902 (52.4)	714 (52.5)	734 (52.6)
BMI	28.30 ± 5.44	26.41 ± 4.10	31.16 ± 6.23
Race and ethnicity			
Chinese	693 (12.5)	162 (11.9)	117 (8.4)
Hispanic	1228 (22.2)	268 (19.7)	381 (27.3)
Non-Hispanic Black	1428 (25.8)	464 (34.1)	313 (22.4)
Non-Hispanic White	2186 (39.5)	466 (34.3)	585 (41.9)
Cigarette smoking			
Never	2817 (50.9)	740 (54.4)	664 (47.6)
Former	2020 (36.5)	420 (30.9)	577 (41.3)
Current	698 (12.6)	200 (14.7)	155 (11.1)
Pack-year of smoking	0.00 (0.00–15.00)	0.00 (0.00–10.04)	0.60 (0.00–21.00)
Asthma	550 (9.9)	132 (9.7)	141 (10.1)
AI-quantified emphysema-like lung (%)	2.02 (0.88–4.04)	1.68 (0.70–3.30)	2.31 (1.06–4.54)
Inflammatory markers			
CRP (mg/dL)	1.89 (0.83–4.16)	1.27 (0.59–3.02)	2.74 (1.22–5.59)
IL-6 (mg/dL)	1.20 (0.78–1.88)	0.91 (0.60–1.40)	1.58 (1.09–2.40)
Type 2 diabetes mellitus	633 (11.4)	83 (6.1)	259 (18.6)
Physical activity (MET, h/wk)	58.25 (28.75–107.00)	71.13 (36.75–124.00)	49.25 (22.94–91.06)
HOMA-IR	2.05 (1.39–3.18)	1.67 (1.22–2.61)	2.52 (1.69–3.92)

Note.—Data are presented as medians with IQRs in parentheses or means ± SDs for continuous variables and numbers with percentages in parentheses for categorical variables. AI = artificial intelligence, BMI = body mass index (calculated by dividing weight in kilograms by height in meters squared), CAC = coronary artery calcium, CRP = C-reactive protein, HOMA-IR = homeostasis model assessment of insulin resistance, IL-6 = interleukin 6, MET = metabolic equivalent of task, TSM = thoracic skeletal muscle.

### Clinically Diagnosed COPD Incidence and Associated Characteristics

During a median follow-up of 18.64 years (IQR, 12.06–19.50 years), 396 participants developed COPD (7.1%), corresponding to an incidence rate of 0.5 per 100 person-years. Among participants with COPD, 219 were male (55.3%) and 177 were female (44.7%). Baseline characteristics by COPD status (event vs no event) are summarized in Table 2.

**Table 2:** Clinical Characteristics in Participants with and without Incidence of Clinically Diagnosed COPD

Characteristic	No Event (*n* = 5139)	COPD Group (*n* = 396)	*P* Value*
CAC CT scan AI output			
TSM mean attenuation (HU)	34.65 ± 7.64	31.59 ± 7.77	<.001
TSM volume (mL)	1178.54 ± 388.71	1167.86 ± 349.58	.56
Emphysema-like lung (%)	1.98 (0.88–3.93)	2.95 (1.30–5.78)	<.001
Age (y)	61.83 ± 10.32	66.92 ± 8.92	<.001
Male sex	2725 (53.0%)	219 (55.3%)	.002
BMI	28.28 ± 5.42	28.62 ± 5.75	.26
Race and ethnicity			.001
Non-Hispanic White	2001 (38.9)	185 (46.7)	
Non-Hispanic Black	1318 (25.6)	110 (27.8)	
Hispanic	1168 (22.7)	60 (15.2)	
Chinese	652 (12.7)	41 (10.4)	
Cigarette smoking			<.001
Never	2721 (52.9)	96 (24.2)	
Former	1842 (35.8)	178 (44.9)	
Current	576 (11.2)	122 (30.8)	
Pack-years of smoking	0.00 (0.00–12.60)	21.75 (0.11–45.00)	<.001
Asthma	485 (9.4)	65 (16.4)	<.001
Inflammatory markers			
CRP (mg/dL)	1.81 (0.81–4.08)	2.70 (1.21–5.06)	<.001
IL-6 (mg/dL)	1.18 (0.76–1.84)	1.48 (1.06–2.32)	<.001
Type 2 diabetes mellitus	579 (11.3)	54 (13.6)	.18
Physical activity (MET, h/wk)	58.58 (29.00–107.50)	50.00 (25.38–100.42)	.15
HOMA-IR	2.04 (1.38–3.15)	2.22 (1.48–3.40)	.15

Note.—Data are presented as medians with IQRs in parentheses or means ± SDs for continuous variables and numbers with percentages in parentheses for categorical variables. AI = artificial intelligence, BMI = body mass index (calculated by dividing weight in kilograms by height in meters squared), CAC = coronary artery calcium, COPD = chronic obstructive pulmonary disease, CRP = C-reactive protein, HOMA-IR = homeostasis model assessment of insulin resistance, IL-6 = interleukin 6, MET = metabolic equivalent of task, TSM = thoracic skeletal muscle.

* *P* values compare COPD versus no COPD, using *t* tests or Mann-Whitney *U* test for continuous variables and χ^2^ or the Fisher exact test for categorical variables.

### Association between Myosteatosis and COPD

**Figure 2:**
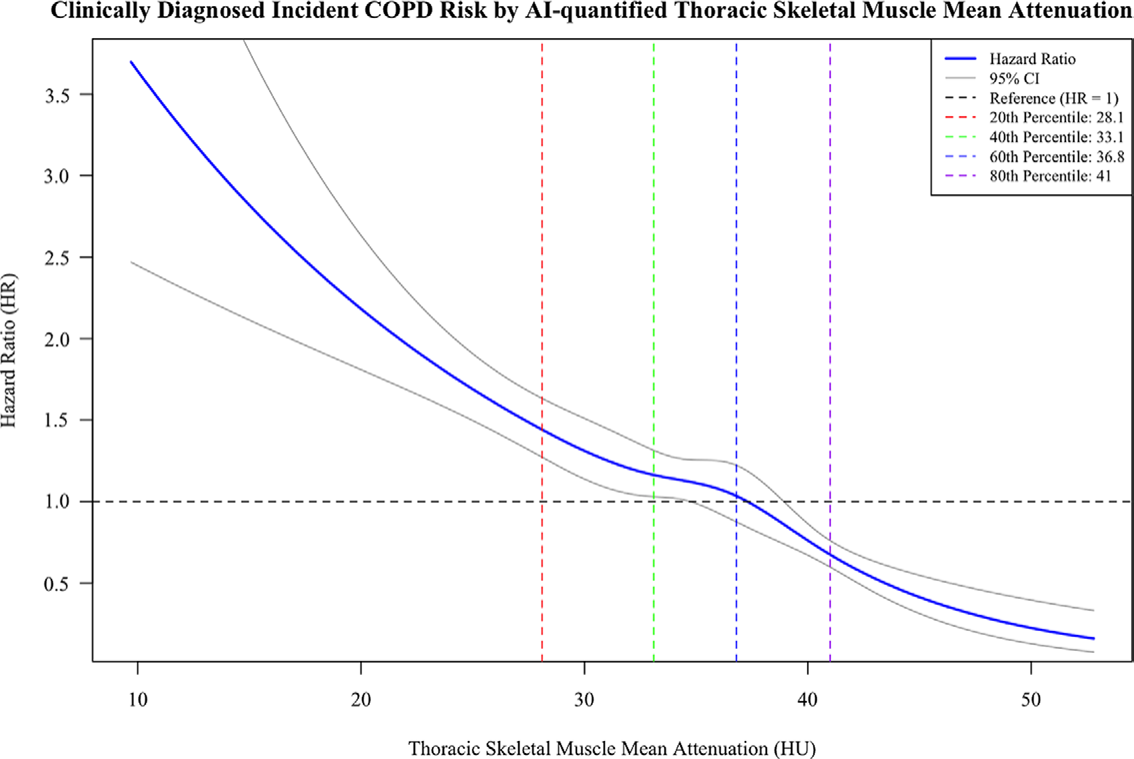
Graph shows Cox regression using restricted cubic splines to model the association between TSM mean attenuation and clinically diagnosed COPD risk, with quantile-spaced knots. AI = artificial intelligence, COPD = chronic obstructive pulmonary disease, TSM = thoracic skeletal muscle.

Univariate analysis comparing the lowest versus highest quartile of TSM attenuation revealed a strong association between myosteatosis and the incidence of clinically diagnosed COPD, with a hazard ratio (HR) of 5.98 (95% CI: 4.14, 8.63). This association remained consistent after adjusting for age, sex, body mass index, race, pack-year smoking, asthma, physical activity, inflammatory factors, and insulin resistance. Following full adjustment, myosteatosis maintained an independent association with the incidence of clinically diagnosed COPD, with an adjusted HR of 2.74 (95% CI: 1.81, 4.16) (Table 3). Furthermore, after adjusting for emphysema-like percentage, the association remained significant, with an HR of 2.50 (95% CI: 1.64, 3.80). The continuous TSM mean attenuation was associated with an HR of 1.06 (95% CI: 1.05, 1.07) for every unit decrease in TSM mean attenuation. Similarly, for every 1 SD decrease in TSM mean attenuation, the HR was 1.55 (95% CI: 1.41, 1.70) (Fig 2).

**Table 3:** Hazards Ratios for Incidence of Clinically Diagnosed COPD by AI-quantified TSM Mean Attenuation and Emphysema-like Lung Quartiles (Comparing Top Quartile vs Bottom Quartile)

Model	Bottom Quartile of AI-quantified TSM Mean Attenuation (Myosteatosis) HR (95% CI)	Top Quartile of AI-quantified Emphysema-like Lung HR (95% CI)	*P* Value*
Model 1 (unadjusted)	5.98 (4.14, 8.63)	2.12 (1.61, 2.78)	<.001
Model 2 (age adjusted)	3.40 (2.31, 5.01)	1.62 (1.23, 2.14)	.002
Model 3 (model 2 + sex, BMI, race and ethnicity†)	3.53 (2.33, 5.34)	1.60 (1.21, 2.13)	.002
Model 4 (model 3 + pack-year smoking)	2.87 (1.89, 4.36)	1.51 (1.14, 2.01)	.01
Model 5 (model 4 + asthma)	2.99 (1.97, 4.54)	1.46 (1.10, 1.94)	.005
Model 6 (model 5 + physical activity)	2.98 (1.97, 4.52)	1.46 (1.10, 1.94)	.005
Model 7 (model 6+ inflammatory index [CRP and IL-6])	2.76 (1.82, 4.20)	1.45 (1.11, 1.96)	.01
Model 8 (model 7 + HOMA-IR)	2.74 (1.81, 4.16)	1.50 (1.12, 2.00)	.02

Note.—BMI = body mass index (calculated by dividing weight in kilograms by height in meters squared), COPD = chronic obstructive pulmonary disease, CRP = C-reactive protein, HOMA-IR = homeostasis model assessment of insulin resistance, HR = hazard ratio, IL-6 = interleukin 6, TSM = thoracic skeletal muscle.

* Wald test comparing the myosteatosis HR (95% CI) and emphysema-like lung HR (95% CI).

^†^ Race and ethnicity groups are Chinese, Black, Hispanic versus White (reference).

**Figure 3:**
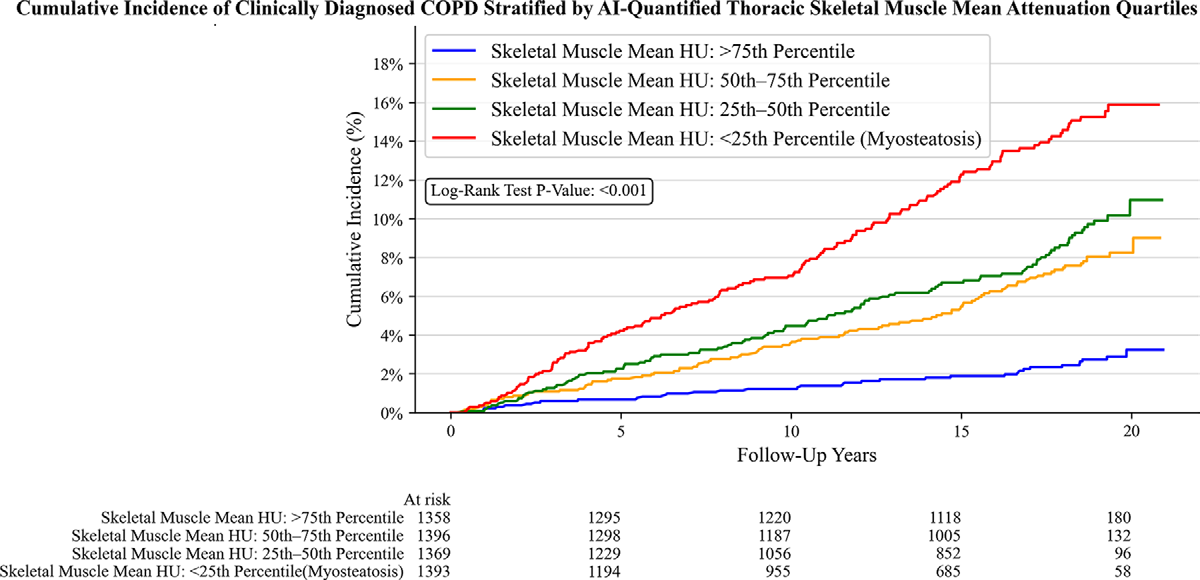
Graph shows cumulative incidence of clinically diagnosed COPD stratified by TSM mean attenuation quartiles. AI = artificial intelligence, COPD = chronic obstructive pulmonary disease, TSM = thoracic skeletal muscle.

The cumulative incidence analysis demonstrated that individuals in the lowest quartile of TSM attenuation (myosteatosis) had a higher incidence of clinically diagnosed COPD, 15.9% (95% CI: 13.6, 18.5), compared with the highest quartile, 3.2% (95% CI: 2.2, 4.7) (*P* < .001) (Fig 3).

### Comparison with Emphysema-like Lung in Cardiac CT

Univariate analysis comparing the highest versus lowest quartile of emphysema-like lung revealed a strong association between emphysema-like lung and incidence of clinically diagnosed COPD, with an HR of 2.12 (95% CI: 1.61, 2.78). This association remained consistent after adjusting for age, sex, body mass index, race, pack-year smoking, asthma, physical activity, inflammatory factors, and insulin resistance. Following full adjustment, emphysema-like lung maintained an independent association with the incidence of clinically diagnosed COPD, with an adjusted HR of 1.50 (95% CI: 1.12, 2.00). Furthermore, after adjusting for TSM mean attenuation, the association remained significant, with an HR of 1.39 (95% CI: 1.04, 1.86).

Notably, Wald test analyses consistently revealed that myosteatosis exhibited stronger predictive power for the incidence of clinically diagnosed COPD compared with emphysema-like lung across all tested models (*P* < .05), suggesting its potential superiority as a risk indicator for COPD development (Table 3). The continuous emphysema-like lung percentage was associated with an HR of 1.13 (95% CI: 1.11, 1.15) for every percentage increase in emphysema-like lung percentage. Similarly, for every SD increase in emphysema-like lung percentage, the HR was 1.46 (95% CI: 1.38, 1.54).

**Figure 4:**
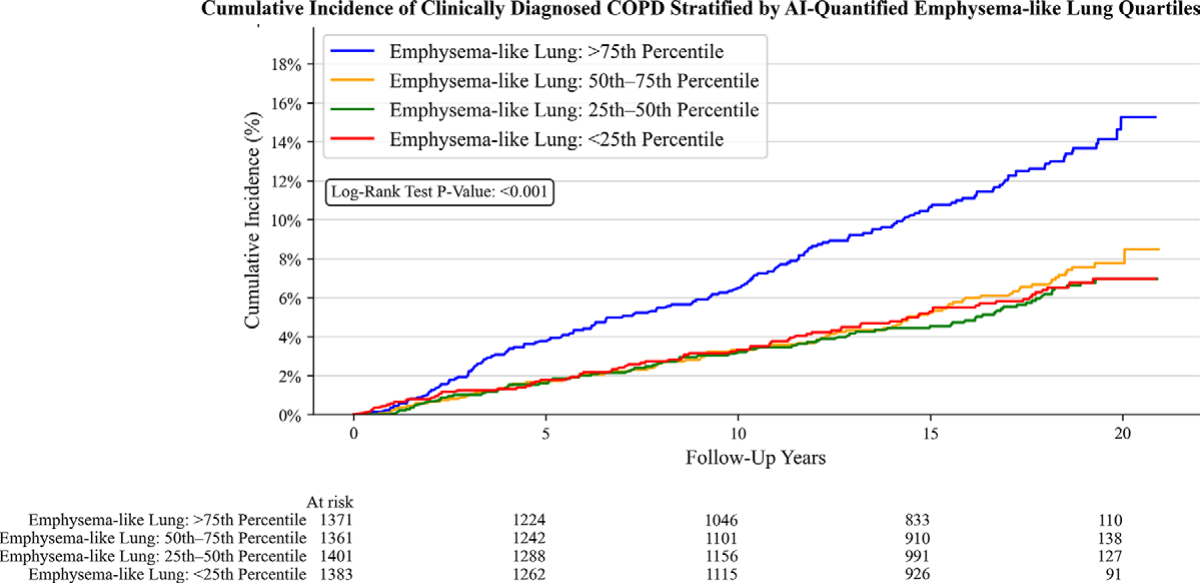
Graph shows cumulative incidence of clinically diagnosed COPD stratified by emphysema-like lung quartiles. AI = artificial intelligence, COPD = chronic obstructive pulmonary disease.

**Figure 5:**
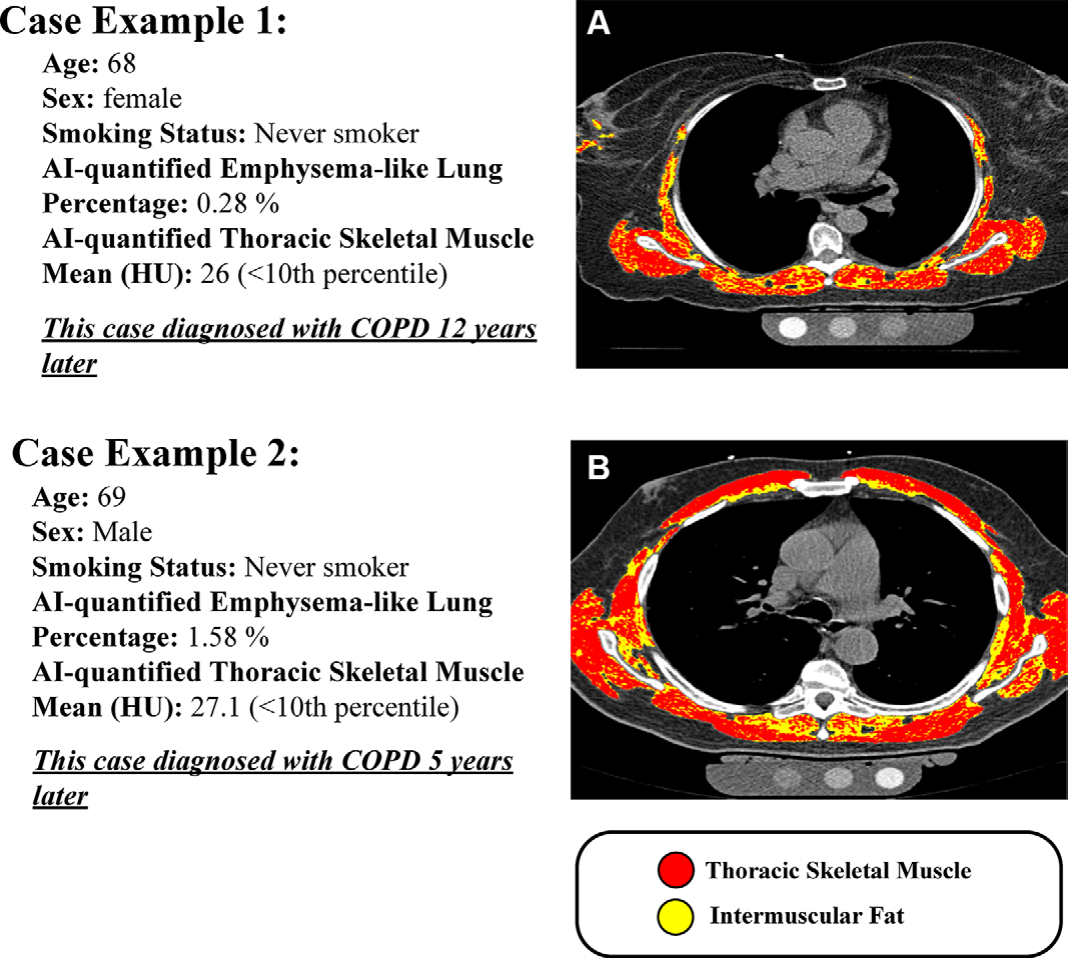
Example images of noncontrast, electrocardiographically gated coronary artery calcium CT (CAC CT) in the axial plane (section thickness, 2.5 mm). AI overlays show thoracic skeletal muscle (red) and intermuscular adipose tissue (yellow) for two MESA participants who never smoked, both with low emphysema-like lung percentage and evidence of myosteatosis, and who developed COPD during follow-up. **(A)** A 68-year-old female participant diagnosed with COPD 12 years after imaging. **(B)** A 69-year-old male participant diagnosed 5 years after imaging. AI = artificial intelligence, COPD = chronic obstructive pulmonary disease, MESA = Multi-Ethnic Study of Atherosclerosis, TSM = thoracic skeletal muscle.

The cumulative incidence analysis demonstrated that individuals in the highest quartile of emphysema-like lung percentage had a higher incidence of clinically diagnosed COPD, 15.3% (95% CI: 12.8, 18.1), compared with the lowest quartile, 7.0% (95% CI: 5.6, 8.7) (*P* < .001) (Fig 4). We present two participants who never smoked with myosteatosis and low emphysema-like lung who developed COPD during follow-up (Fig 5).

### Subgroup Analyses of Myosteatosis and COPD Risk

Subgroup analyses demonstrated associations between myosteatosis and the incidence of clinically diagnosed COPD across age categories, sex, obesity status, smoking history, passive smoking exposure, physical activity levels, insulin resistance status, and emphysema-like lung percentage stratified by median values (Table S1).

### Sensitivity Analyses of Myosteatosis and COPD Risk

In sensitivity analyses, the results remained consistent after excluding participants who experienced COPD during the first 2 years of follow-up (*n* = 33) and participants with asthma at baseline (*n* = 549) (Table S2).

### Interaction Analyses

In the fully adjusted model including both attenuation × age and attenuation × pack-years terms, neither interaction was significant (Wald *P* = .48 and *P* = .69, respectively). Moreover, the likelihood-ratio test comparing this model to the base model yielded a χ^2^ equaling 0.69 and a *P* value of .71, indicating that adding these interaction terms did not improve model fit.

## Discussion

To the best of our knowledge, this is the first study to investigate the association between AI-quantified TSM myosteatosis and future COPD diagnosis. In this prospective study of 5535 MESA participants followed for 20 years, AI-quantified myosteatosis from baseline CAC CT scans strongly predicted incident clinically diagnosed COPD. After adjusting for age, sex, body mass index, race, smoking history, asthma, physical activity, inflammatory markers, and insulin resistance, participants with myosteatosis (lowest quartile of TSM mean attenuation) demonstrated a 2.74-fold increased risk of developing COPD compared with those in the highest quartile. Notably, myosteatosis outperformed AI-measured emphysema-like lung as a predictor, with adjusted HRs of 2.74 versus 1.50, respectively (*P *= .02). This association remained robust across subgroups stratified by age, sex, obesity, smoking status, physical activity, insulin resistance, and emphysema-like lung levels and persisted in sensitivity analyses excluding early COPD cases and participants with baseline asthma.

Although numerous studies have examined muscle abnormalities in established COPD, research investigating myosteatosis as a predictor of COPD in asymptomatic individuals remains limited. Previous investigations have consistently demonstrated elevated skeletal muscle fat infiltration in patients with COPD compared with healthy controls, with these alterations strongly correlating with disease severity and adverse clinical outcomes (18–20). Persson et al (18) reported that increased muscle fat infiltration in patients with COPD was associated with heightened systemic inflammation and impaired muscle bioenergetic function. Qiao et al (3) found that lower pectoralis muscle attenuation predicted respiratory failure during acute exacerbations, and Park et al (19) demonstrated that reduced intercostal muscle attenuation predicted accelerated lung function decline. Although these studies highlight the prognostic significance of myosteatosis in advanced disease stages, the critical gap remains in understanding its role in preclinical COPD pathogenesis.

The robust association between myosteatosis and the incidence of clinically diagnosed COPD observed in our study suggests several potential mechanistic pathways. Although prior studies reported significantly elevated inflammatory markers, supporting the role of systemic inflammation as a key mediator (18,26), we did not find strong correlations between interleukin 6 or C-reactive protein with myosteatosis in MESA participants. This complex relationship may be partially explained by the limitations of spot measurements of these serum biomarkers, which can fluctuate considerably over short periods, unlike the relatively stable nature of myosteatosis.

Another plausible pathway involves metabolic dysfunction. Intramuscular fat accumulation has been linked to insulin resistance and disturbances in energy metabolism—conditions that may contribute to oxidative stress in pulmonary tissues (21,27). These systemic effects may impair tissue repair and promote airway remodeling. Prior evidence from the MESA cohort linked low muscle attenuation to impaired glucose regulation and increased diabetes risk (28). Existing literature suggests that diabetes and prediabetes may elevate COPD risk via mechanisms such as systemic inflammation, oxidative damage, glucotoxicity, and autonomic imbalance (7). Notably, the substantial attenuation in the predictive value of myosteatosis following age adjustment reflects the complex interplay between aging and muscle quality deterioration in COPD pathogenesis.

In this study, we found that both AI-measured emphysema-like lung in CAC CT scans and myosteatosis independently predicted COPD, even after adjusting for smoking status. Our findings suggest that myosteatosis may be a more potent predictor of COPD development than emphysema-like lung, reflecting its role as an indicator of early pathophysiologic changes. The deterioration in muscle quality is associated with metabolic alterations and systemic inflammation, which may contribute to and accelerate lung damage.

Unlike traditional studies that rely on single-section or region of interest–based analysis of specific muscle groups, our approach leverages deep learning algorithms to perform comprehensive segmentation and measurement across all thoracic skeletal muscle visible in CAC CT scans, improving reproducibility and anatomic coverage.

Some limitations should be considered in our study. First, although we adjusted for multiple potential confounders, residual confounding by unmeasured factors cannot be excluded in this observational study design. Second, muscle assessment was limited to a single time point, preventing analysis of temporal changes in muscle quality and their relationship to COPD risk. Third, a significant technical limitation involves the lack of standardized CT parameters for myosteatosis measurements. Fourth, the accuracy of muscle quality assessments may be affected by variations in CT parameters such as tube voltage, tube current, section thickness, and reconstruction algorithms, particularly when using narrow attenuation thresholds. Fifth, it is important to note that the MESA baseline dataset lacked spirometry data for assessing asymptomatic patients with COPD. Previous studies have shown that COPD prevalence among asymptomatic individuals ranges from approximately 1.5% to 13.2%, depending on the population studied, risk factors present, and diagnostic criteria used (29–31). Additionally, CAC CT scans do not cover the upper lung regions where emphysema is most commonly observed, which is an important consideration when interpreting these findings. Sixth, our COPD outcome definition relies on hospital discharge records, which limits our findings to COPD cases severe enough to require hospitalization and may miss milder cases managed in outpatient settings. Finally, our study used both electron-beam CT and multidetector row CT scans from the MESA CAC CT scan protocol, and, given that electron-beam CT scanners are no longer manufactured, future validation studies using modern multidetector row CT scanners with higher spatial resolution will be necessary to confirm our findings.

In conclusion, AI-quantified myosteatosis from CAC CT scans represents a powerful independent predictor of incident COPD that outperforms emphysema-like lung measurements. Future research should incorporate longitudinal muscle quality assessments to clarify temporal relationships with subclinical lung function decline, use full chest CT with spirometry while excluding patients with spirometry-diagnosed COPD at baseline, and explore mechanistic pathways linking myosteatosis to COPD development. Additionally, extending opportunistic body composition analysis to nonthoracic muscle groups using full-body or abdominal CT or MRI protocols will help clarify whether myosteatosis outside the thoracic cavity similarly predicts incident COPD and distinguish generalized preclinical muscle fat infiltration from early respiratory muscle wasting. These advances may identify novel therapeutic targets and enable earlier preventive interventions in high-risk populations.

## Supplemental Files

Appendices S1-S2, Tables S1-S2, Figure S1

Conflicts of Interest

## References

[1] Smith BM , Kirby M , Hoffman EA , et al ; MESA Lung, CanCOLD, and SPIROMICS Investigators . Association of dysanapsis with chronic obstructive pulmonary disease among older adults . JAMA 2020 ; 323 ( 22 ): 2268 – 2280 . 32515814 10.1001/jama.2020.6918 PMC7284296

[2] Safiri S , Carson-Chahhoud K , Noori M , et al . Burden of chronic obstructive pulmonary disease and its attributable risk factors in 204 countries and territories, 1990-2019: results from the Global Burden of Disease Study 2019 . BMJ 2022 ; 378 : e069679 . 35896191 10.1136/bmj-2021-069679 PMC9326843

[3] Qiao X , Hou G , Kang J , Wang QY , Yin Y . CT attenuation and cross-sectional area of the pectoralis are associated with clinical characteristics in chronic obstructive pulmonary disease patients . Front Physiol 2022 ; 13 : 833796 . 35721549 10.3389/fphys.2022.833796 PMC9205603

[4] Vivodtzev I , Moncharmont L , Tamisier R , et al . Quadriceps muscle fat infiltration is associated with cardiometabolic risk in COPD . Clin Physiol Funct Imaging 2018 ; 38 ( 5 ): 788 – 797 . 29105276 10.1111/cpf.12481

[5] Goodpaster BH , Kelley DE , Thaete FL , He J , Ross R . Skeletal muscle attenuation determined by computed tomography is associated with skeletal muscle lipid content . J Appl Physiol (1985) 2000 ; 89 ( 1 ): 104 – 110 . 10904041 10.1152/jappl.2000.89.1.104

[6] Taaffe DR , Henwood TR , Nalls MA , Walker DG , Lang TF , Harris TB . Alterations in muscle attenuation following detraining and retraining in resistance-trained older adults . Gerontology 2009 ; 55 ( 2 ): 217 – 223 . 19060453 10.1159/000182084 PMC2756799

[7] Su J , Li M , Wan X , et al . Associations of diabetes, prediabetes and diabetes duration with the risk of chronic obstructive pulmonary disease: A prospective UK Biobank study . Diabetes Obes Metab 2023 ; 25 ( 9 ): 2575 – 2585 . 37248816 10.1111/dom.15142

[8] Su B , Liu T , Fan H , et al . Inflammatory markers and the risk of chronic obstructive pulmonary disease: a systematic review and meta-analysis . PLoS One 2016 ; 11 ( 4 ): e0150586 . 27104349 10.1371/journal.pone.0150586 PMC4841528

[9] Li S , Zhang T , Yang H , et al . Metabolic syndrome, genetic susceptibility, and risk of chronic obstructive pulmonary disease: The UK Biobank Study . Diabetes Obes Metab 2024 ; 26 ( 2 ): 482 – 494 . 37846527 10.1111/dom.15334

[10] Pickhardt PJ . Value-added opportunistic CT screening: state of the art . Radiology 2022 ; 303 ( 2 ): 241 – 254 . [Published correction appears in Radiology 2022;303(3):E41.] 35289661 10.1148/radiol.211561 PMC9083232

[11] Naghavi M , Reeves AP , Atlas K , et al . Artificial intelligence applied to coronary artery calcium scans (AI-CAC) significantly improves cardiovascular events prediction . NPJ Digit Med 2024 ; 7 ( 1 ): 309 . 39501071 10.1038/s41746-024-01308-0 PMC11538462

[12] Naghavi M , Atlas K , Zhang C , et al . AI Measurement Of Myosteatosis In Cardiac CT Predicts Atrial Fibrillation And Heart Failure: An AI-CVD Study . J Cardiovasc Comput Tomogr 2025 ; 19 ( 4 ): S70 .

[13] Naghavi M , Reeves A , Atlas K , et al . AI-enabled Cardiac Chambers Volumetry and Calcified Plaque Characterization in Coronary Artery Calcium (CAC) Scans (AI-CAC) . Significantly Improves on Agatston CAC Score for Predicting All Cardiovascular Events: The Multi-Ethnic Study of Atherosclerosis . Res Sq [preprint] 2024 Jun 20:rs.3.rs-4433105 .

[14] Naghavi M , De Oliveira I , Mao SS , et al . Opportunistic AI-enabled automated bone mineral density measurements in lung cancer screening and coronary calcium scoring CT scans are equivalent . Eur J Radiol Open 2023 ; 10 : 100492 . 37214544 10.1016/j.ejro.2023.100492 PMC10196960

[15] Naghavi M , Yankelevitz D , Reeves AP , et al . AI-enabled left atrial volumetry in coronary artery calcium scans (AI-CACTM) predicts atrial fibrillation as early as one year, improves CHARGE-AF, and outperforms NT-proBNP: The multi-ethnic study of atherosclerosis . J Cardiovasc Comput Tomogr 2024 ; 18 ( 4 ): 383 – 391 . 38653606 10.1016/j.jcct.2024.04.005 PMC11216863

[16] Naghavi M , Reeves A , Budoff M , et al . AI-enabled cardiac chambers volumetry in coronary artery calcium scans (AI-CACTM) predicts heart failure and outperforms NT-proBNP: the multi-ethnic study of atherosclerosis . J Cardiovasc Comput Tomogr 2024 ; 18 ( 4 ): 392 – 400 . 38664073 10.1016/j.jcct.2024.04.006 PMC11216890

[17] Naghavi M , Reeves AP , Atlas KC , et al . AI-Enabled CT Cardiac Chamber Volumetry Predicts Atrial Fibrillation and Stroke Comparable to MRI . JACC Adv 2024 ; 3 ( 11 ): 101300 . 39741645 10.1016/j.jacadv.2024.101300 PMC11686054

[18] Persson HL , Sioutas A , Kentson M , et al . Skeletal myosteatosis is associated with systemic inflammation and a loss of muscle bioenergetics in stable COPD . J Inflamm Res 2022 ; 15 : 4367 – 4384 . 35937916 10.2147/JIR.S366204 PMC9355337

[19] Park MJ , Cho JM , Jeon KN , et al . Mass and fat infiltration of intercostal muscles measured by CT histogram analysis and their correlations with COPD severity . Acad Radiol 2014 ; 21 ( 6 ): 711 – 717 . 24809313 10.1016/j.acra.2014.02.003

[20] Jeon YJ , Han S , Park GM , et al . Intramuscular and intermuscular abdominal fat infiltration in copd: a propensity score matched study . Int J Chron Obstruct Pulmon Dis 2021 ; 16 : 1989 – 1999 . 34262269 10.2147/COPD.S312888 PMC8275100

[21] Jung M , Rieder H , Reisert M , et al . Association between myosteatosis and impaired glucose metabolism: A deep learning whole‐body magnetic resonance imaging population phenotyping approach . J Cachexia Sarcopenia Muscle 2024 ; 15 ( 5 ): 1750 – 1760 . 39009381 10.1002/jcsm.13527 PMC11446675

[22] Bild DE , Bluemke DA , Burke GL , et al . Multi-ethnic study of atherosclerosis: objectives and design . Am J Epidemiol 2002 ; 156 ( 9 ): 871 – 881 . 12397006 10.1093/aje/kwf113

[23] Gevenois PA , de Maertelaer V , De Vuyst P , Zanen J , Yernault JC . Comparison of computed density and macroscopic morphometry in pulmonary emphysema . Am J Respir Crit Care Med 1995 ; 152 ( 2 ): 653 – 657 . 7633722 10.1164/ajrccm.152.2.7633722

[24] Wasserthal J , Breit HC , Meyer MT , et al . TotalSegmentator: robust segmentation of 104 anatomic structures in CT images . Radiol Artif Intell 2023 ; 5 ( 5 ): e230024 . 37795137 10.1148/ryai.230024 PMC10546353

[25] Araki T , Nishino M , Zazueta OE , et al . Paraseptal emphysema: prevalence and distribution on CT and association with interstitial lung abnormalities . Eur J Radiol 2015 ; 84 ( 7 ): 1413 – 1418 . 25868675 10.1016/j.ejrad.2015.03.010 PMC4450117

[26] Aro R , Meriläinen S , Sirniö P , et al . Sarcopenia and myosteatosis are associated with neutrophil to lymphocyte ratio but not glasgow prognostic score in colorectal cancer patients . J Clin Med 2022 ; 11 ( 9 ): 2656 . 35566781 10.3390/jcm11092656 PMC9104763

[27] Savage DB , Watson L , Carr K , et al . Accumulation of saturated intramyocellular lipid is associated with insulin resistance . J Lipid Res 2019 ; 60 ( 7 ): 1323 – 1332 . 31048405 10.1194/jlr.M091942 PMC6602127

[28] Gold RS , Unkart JT , Larsen BA , et al . Association of abdominal muscle area and density with glucose regulation: The multi‐ethnic study of atherosclerosis (MESA) . Diabetes Metab Res Rev 2022 ; 38 ( 2 ): e3488 . 34328704 10.1002/dmrr.3488 PMC8800952

[29] Yin X , Zheng Z , Dong Y , et al . Comparison of newly diagnosed COPD patients and the non-COPD residents in Shanghai Minhang District . Front Public Health 2023 ; 11 : 1102509 . 36935678 10.3389/fpubh.2023.1102509 PMC10014998

[30] Sansores RH , Velázquez-Uncal M , Pérez-Bautista O , Villalba-Caloca J , Falfán-Valencia R , Ramírez-Venegas A . Prevalence of chronic obstructive pulmonary disease in asymptomatic smokers . Int J Chron Obstruct Pulmon Dis 2015 ; 10 : 2357 – 2363 . 26586941 10.2147/COPD.S91742 PMC4636090

[31] Fortis S . Should we consider screening spirometry in individuals who are "asymptomatic"? . Ann Am Thorac Soc 2022 ; 19 ( 8 ): 1268 – 1269 . 35913464 10.1513/AnnalsATS.202205-374ED PMC9353965

