# Supplementary material for: Artificial Intelligence–derived Measurements of Myosteatosis from Coronary Artery Calcium CT Scans to Predict COPD: The Multi-Ethnic Study of Atherosclerosis
Source: Radiol Cardiothorac Imaging. 2026 Jan 29;8(1):e250205. doi: 10.1148/ryct.250205 (PMC12951201; doi:10.1148/ryct.250205)
Supplement: Appendices S1-S2, Tables S1-S2, Figure S1 [file ryct250205suppa1.pdf]

Supplemental materials undergo peer review but are not copyedited, and may include typographical errors.

©The Author(s) 2026. Published by the Radiological Society of North America under a CC BY 4.0 license.
10.1148/ryct.250205

Supplementary Materials

Appendix S1. AI-CVD Components

Figure S1. AI-CVD Components Diagram

Appendix S2: Imaging Protocol

Table S1. Subgroup Analysis for Incidence of Clinically Diagnosed COPD by AI-Quantified TSM Mean Attenuation Quartiles

Table S2. Sensitivity Analysis for Incidence of Clinically Diagnosed COPD by AI-Quantified TSM Mean Attenuation Quartiles.

Supplemental materials undergo peer review but are not copyedited, and may include typographical errors.

Appendix S1: AI-CVD Components

AI-CVD™:

A Comprehensive Paradigm Shift in CVD Risk Assessment

AI-CVD™ extracts opportunistic screening information beyond the heart, just like AI-CAC extracts opportunistic screening information beyond the coronaries. This innovative approach significantly enhances CVD risk stratification by integrating imaging disease measures. These measures are shown in Figure 11 and described in detail below, enabling a multidimensional evaluation of cardiometabolic health. AI-CVD™ thus emerges as the most comprehensive tool that extends well beyond CHD to predict HF, AF, stroke, total CVD, CVD death, and overall mortality. LV volume and mass are among the strongest predictors of total CVD events as well as incident HF.

Key Components of AI-CVD Beyond the CAC Score Developed by HeartLung:

1. Bone Mineral Density (BMD): Osteoporosis and CVD Risk

Thoracic vertebral bone density, measurable in routine CAC scans, provides an important marker for osteoporosis(1,2). Low BMD is independently associated with increased CVD risk and all-cause mortality, potentially mediated by shared inflammatory and metabolic pathways. Incorporating BMD into CVD risk assessments ensures early detection of both osteoporosis and its cardiovascular implications, enabling timely interventions. HeartLung has received FDA clearance for this component of AI-CVD under AutoBMD AI. (See FDA 510k letter attached)

2. Thoracic Aortic Calcification (TAC): Stroke and CVD Prediction

TAC, an often-overlooked finding in CAC scans, predicts stroke and CVD mortality with greater precision when combined with traditional risk factors (3). As a marker of systemic

Supplemental materials undergo peer review but are not copyedited, and may include typographical errors.

atherosclerosis, TAC enhances risk stratification for cerebrovascular events, broadening the utility of CAC scans.

3. Aortic Valve Calcification (AVC): Identifying Aortic Stenosis Risk

AVC, visible in CAC scans, predicts aortic stenosis and its associated cardiovascular outcomes (4). Early identification of AVC facilitates closer monitoring and timely surgical or medical interventions, reducing morbidity and mortality from valve-related complications.

4. Cardiac Chamber Volumetry: Heart Failure and Atrial Fibrillation

AI-driven analysis of cardiac chamber volumes—including the left atrium (LA), left ventricle (LV), right atrium (RA), and right ventricle (RV) provides actionable insights into heart failure and atrial fibrillation risk. Enlarged LA volume strongly predicts AF and stroke, while LV hypertrophy correlates with HF and coronary artery disease. These measurements add critical dimensions to CAC scans, improving predictive accuracy (5–8).

5. Liver Fat: Cardiometabolic Risk

Fatty liver, detected opportunistically in CAC scans, correlates with metabolic syndrome, diabetes, and heightened CVD risk (9). By quantifying liver fat, AI-CVD™ identifies individuals at risk for cardiometabolic events, offering opportunities for early lifestyle or pharmacological interventions (10).

6. Visceral Fat: Metabolic and Inflammatory Burden

Visceral fat—a major driver of systemic inflammation and insulin resistance—is measurable in CAC scans. Increased visceral fat volume is a powerful predictor of metabolic syndrome, type 2 diabetes, and CVD. AI-CVD's ability to quantify visceral fat improves risk stratification for these conditions.

7. Emphysema: COPD and CVD Risk

Supplemental materials undergo peer review but are not copyedited, and may include typographical errors.

Pulmonary emphysema, visible in chest CT scans, is linked to chronic obstructive pulmonary disease (COPD) and increased cardiovascular mortality. Including emphysema scoring in AI-CVD highlights the interplay between pulmonary and cardiovascular health, fostering integrated care approaches.

8. Myosteatosis: Muscle Quality as a New Cardiometabolic Biomarker

Myosteatosis, characterized by fat infiltration into skeletal muscle, is an emerging biomarker of systemic metabolic dysfunction (11). AI-driven measurement of thoracic skeletal muscle density from CAC scans has shown strong predictive value for HF, AF, CHD, and all-cause mortality. Recent studies demonstrate that combining myosteatosis with CAC scores amplifies risk prediction, particularly for males, making it a critical addition to AI-CVD.

9. Calcium Score and Calcified Plaque Characterization

The value of a CAC score is well established. With this AI-CVD component we report not only the weighted Agatston CAC score but also the area, density, location, distribution and shape of each calcified plaque and will be able to monitor changes in each plaque over time while reporting new plaques in the same artery or another artery.

Supplemental materials undergo peer review but are not copyedited, and may include typographical errors.

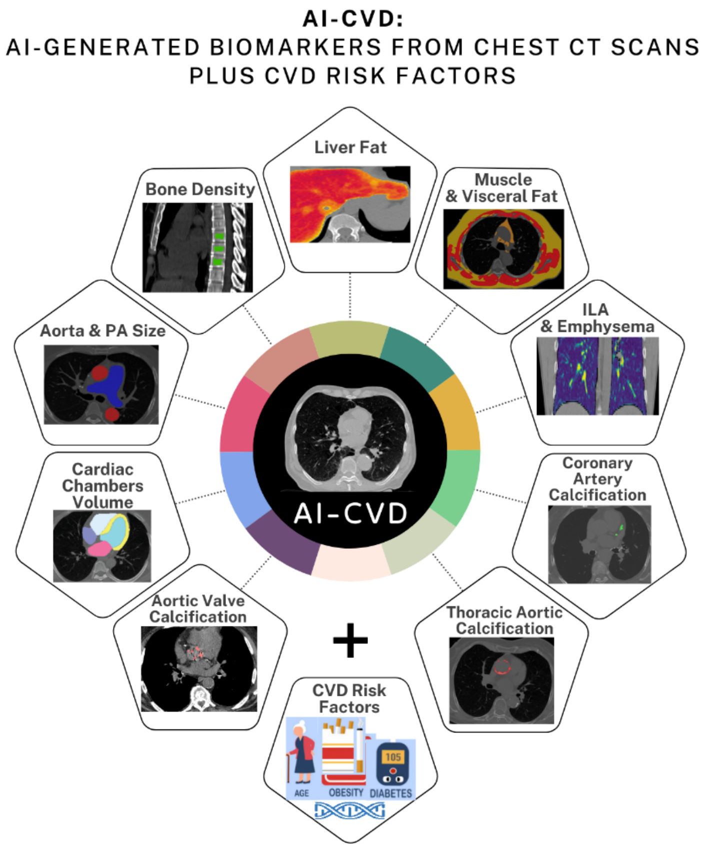

Figure S1. AI-CVD Components Diagram

Supplemental materials undergo peer review but are not copyedited, and may include typographical errors.

Appendix S2: Imaging Protocol

Baseline cardiac CT scanning employed two technologies: Three centers (New York, Illinois, and California) utilized electron-beam CT with image acquisition gated at 50% of the R-R interval, while the remaining sites (Maryland, Minnesota, and North Carolina) used multi-detector row CT with gating at 80% of the R-R interval. Each scan was performed from the carina to below the apex of the heart. The scans yielded an average of 44 images per participant. To ensure consistency across sites, image reconstruction parameters were standardized: 2.5 mm slice thickness, 512×512 matrix, 350 mm field of view (encompassing the entire chest width), and 0.68 mm in-plane pixel spacing.

Supplemental materials undergo peer review but are not copyedited, and may include typographical errors.

Table S1. Subgroup Analysis for Incidence of Clinically Diagnosed COPD by AI-Quantified TSM Mean Attenuation Quartiles

Category			Event / Sample	Unadjusted HR (95% CI)	Adjusted HR (95% CI)
**Age**					
	**Age ≥60 years**				
		<25% quartile (Myosteatosis)	142/1089	3.55 (2.11–5.97)	3.38 (1.98–5.76)
		25%–50%	83/951	2.17 (1.26–3.72)	1.98 (1.15–3.40)
		50%–75%	67/740	1.95 (1.12–3.39)	1.85 (1.06–3.24)
		>75%	18/388	Reference	Reference
	**Age <60 years**				
		<25% quartile (Myosteatosis)	17/307	3.85 (2.09–7.13)	3.37 (1.68–6.76)
		25%–50%	23/428	3.35 (1.88–5.94)	3.23 (1.76–5.93)
		50%–75%	29/660	2.90 (1.67–5.03)	2.83 (1.60–5.00)
		>75%	17/972	Reference	Reference
**Sex**					
	**Male**				
		<25% quartile (Myosteatosis)	89/662	6.48 (3.94–10.64)	3.44 (1.96–6.04)
		25%–50%	57/655	3.64 (2.16–6.12)	2.05 (1.18–3.55)
		50%–75%	54/670	2.99 (1.77–5.04)	2.27 (1.33–3.89)
		>75%	19/646	Reference	Reference
	**Female**				
		<25% quartile (Myosteatosis)	70/734	5.53 (3.21–9.52)	2.39 (1.28–4.46)
		25%–50%	49/724	3.53 (2.01–6.21)	2.06 (1.14–3.75)
		50%–75%	42/730	2.79 (1.57–4.97)	1.89 (1.05–3.41)
		>75%	16/714	Reference	Reference
**Obesity**					
	**Obese (BMI ≥30 kg//m^2)**				
		<25% quartile (Myosteatosis)	72/709	8.21 (3.00–22.49)	3.87 (1.36–10.98)
		25%–50%	26/448	4.12 (1.44–11.82)	2.69 (0.93–7.83)
		50%–75%	25/355	4.61 (1.60–13.25)	3.45 (1.19–10.00)
		>75%	4/245	Reference	Reference
	**Non-obese (BMI <30 kg/m^2)**				
		<25% quartile (Myosteatosis)	87/687	3.79 (2.78–5.17)	2.11 (1.50–2.98)
		25%–50%	80/931	2.26 (1.66–3.07)	1.55 (1.12–2.16)
		50%–75%	71/1045	1.64 (1.20–2.24)	1.41 (1.02–1.95)
		>75%	31/1115	Reference	Reference
**Smoking status**					
	**≥20 pack-year**				
		<25% quartile (Myosteatosis)	83/364	3.87 (2.30–6.54)	2.78 (1.54–5.01)
		25%–50%	58/316	2.76 (1.60–4.74)	2.07 (1.17–3.66)
		50%–75%	48/267	2.38 (1.37–4.14)	2.18 (1.24–3.83)
		>75%	17/201	Reference	Reference
	**<20 pack-year**				
		<25% quartile (Myosteatosis)	76/1032	6.44 (3.85–10.78)	2.75 (1.54–4.92)
		25%–50%	48/1063	3.48 (2.02–5.98)	1.89 (1.07–3.34)
		50%–75%	48/1133	2.97 (1.73–5.11)	2.01 (1.15–3.49)
		>75%	18/1159	Reference	Reference
**Passive smoking***					
	**Never**				
		<25% quartile (Myosteatosis)	71/741	12.19 (5.60–26.54)	3.74 (1.62–8.60)
		25%–50%	41/719	6.24 (2.80–13.92)	2.85 (1.25–6.50)
		50%–75%	35/726	4.77 (2.12–10.73)	2.89 (1.27–6.57)
		>75%	7/625	Reference	Reference
	**Ever**				

Supplemental materials undergo peer review but are not copyedited, and may include typographical errors.

**<25% quartile (Myosteatosis)**	47/468	9.25 (4.18–20.47)	3.54 (1.47–8.57)
25%–50%	32/448	5.88 (2.59–13.31)	2.77 (1.18–6.54)
50%–75%	22/458	3.65 (1.56–8.55)	2.21 (0.93–5.28)
>75%	7/503	Reference	Reference
**Physical activity**			
**High activity (≥58.25 MET-h/week)**			
<25% quartile (Myosteatosis)	49/615	4.51 (2.63–7.75)	2.73 (1.48–5.04)
25%–50%	47/654	3.73 (2.16–6.42)	2.47 (1.38–4.41)
50%–75%	45/700	3.03 (1.75–5.23)	2.40 (1.37–4.21)
>75%	17/561	Reference	Reference
**Low activity (<58.25 MET-h/week)**			
<25% quartile (Myosteatosis)	110/781	6.55 (3.93–10.94)	3.77 (2.13–6.68)
25%–50%	59/725	3.29 (1.92–5.64)	2.20 (1.25–3.86)
50%–75%	51/700	2.68 (1.55–4.64)	2.07 (1.18–3.63)
>75%	18/799	Reference	Reference
**Insulin resistance (HOMA-IR)**			
**Insulin-resistant (HOMA-IR ≥2.5)**			
<25% quartile (Myosteatosis)	76/705	5.42 (2.80–10.49)	2.78 (1.35–5.70)
25%–50%	44/552	3.64 (1.83–7.24)	2.35 (1.15–4.78)
50%–75%	34/465	2.97 (1.47–6.01)	2.15 (1.05–4.41)
>75%	10/369	Reference	Reference
**Non-insulin-resistant (HOMA-IR <2.5)**			
<25% quartile (Myosteatosis)	83/691	6.40 (4.09–10.02)	2.20 (1.52–3.18)
25%–50%	62/827	3.48 (2.19–5.55)	1.33 (0.93–1.91)
50%–75%	62/935	2.85 (1.79–4.53)	1.51 (1.06–2.13)
>75%	25/991	Reference	Reference
**AI-quantified emphysema-like lung (%)**			
**High emphysema (≥1.32% for females and ≥3.16% for males)**			
<25% quartile (Myosteatosis)	110/759	7.20 (4.26–12.18)	4.10 (2.29–7.35)
25%–50%	64/709	3.98 (2.30–6.88)	2.55 (1.44–4.53)
50%–75%	49/696	2.71 (1.54–4.76)	2.07 (1.16–3.67)
>75%	16/575	Reference	Reference
**Low emphysema (<1.32% for females and <3.16% for males)**			
<25% quartile (Myosteatosis)	49/637	4.16 (2.44–7.07)	2.41 (1.31–4.42)
25%–50%	42/670	3.01 (1.75–5.18)	1.98 (1.11–3.53)
50%–75%	47/704	3.04 (1.78–5.18)	2.34 (1.35–4.05)
>75%	19/785	Reference	Reference

The Cox models were adjusted for age, sex, ethnicity, BMI, pack-year smoking, asthma, physical activity, inflammatory index (CRP + IL-6), and insulin resistance. BMI: Body Mass Index, CI: Confidence Interval, COPD: Chronic Obstructive Pulmonary Disease, HR: Hazard Ratio, HOMA-IR: Homeostasis Model Assessment of Insulin Resistance, TSM: Thoracic Skeletal Muscle.

*The analysis was done just on never-smokers and former smokers.

Supplemental materials undergo peer review but are not copyedited, and may include typographical errors.

Table S2. Sensitivity Analysis for Incidence of Clinically Diagnosed COPD by AI-Quantified TSM Mean Attenuation Quartiles.

Category	Event / Sample	Unadjusted HR (95%CI)	Adjusted HR (95%CI)
**Excluding the first two years of follow-up (n= 45)**			
<25% quartile (Myosteatosis)	141/1378	6.41 (4.32–9.52)	3.57 (2.29–5.57)
25%-50%	96/1369	3.85 (2.56–5.81)	2.50 (1.62–3.85)
50%-75%	84/1388	2.99 (1.97–4.53)	2.29 (1.49–3.50)
> 75%	30/1355	Reference	Reference
**Excluding the participants with asthma at baseline (n=550)**			
<25% quartile (Myosteatosis)	129/1255	4.84 (3.33–7.04)	2.58 (1.68–3.96)
25%-50%	84/1246	2.82 (1.90–4.18)	1.76 (1.16–2.67)
50%-75%	83/1256	2.51 (1.69–3.72)	1.94 (1.29–2.90)
> 75%	35/1228	Reference	Reference

The Cox models were adjusted for age, sex, ethnicity, BMI, pack year smoking, asthma, physical activity, Inflammatory index (CRP + IL-6), and Insulin resistance. BMI: Body Mass Index, CI: Confidence Interval, HR: Hazard Ratio
